# Pulmonary Cytology in Transformation

**DOI:** 10.1159/acy/abjag007

**Published:** 2026-09-05

**Authors:** Jumpei Kashima, Magdalena M. Brune, Nikolaus Deigendesch, Spasenija Savic Prince, Obinna Chijioke, Eva Darai-Ramqvist, Lukas Bubendorf

**Affiliations:** a Institute of Medical Genetics and Pathology, University Hospital Basel, Basel, Switzerland; b Department of Pathology and Molecular Pathology, University Hospital Zurich, Zurich, Switzerland; c Department of Pathology and Cancer Diagnostics, Karolinska University Hospital, Stockholm, Sweden

**Keywords:** Immunocytochemistry, Proteomics, Artificial intelligence, Digital cytology

## Abstract

**Background:**

Traditionally, pulmonary cytology has been used to diagnose and screen for a variety of respiratory diseases, including lung cancer. Owing to its rapid turnaround time compared with histological diagnosis, therapeutic decisions may occasionally need to be made based on cytological findings alone. In the era of precision medicine, however, biomarker assessment and molecular testing have become increasingly important, particularly in the management of lung cancer. In addition, the digitalization of pathological slides has facilitated the development of artificial intelligence (AI)-based diagnostic tools. The utility of these emerging technologies in pulmonary cytology is currently being actively investigated and continues to evolve, overcoming challenges including low input of tumor cells and the three-dimensional nature of cytological specimens.

**Summary:**

This review summarizes recent advances in pulmonary cytology, focusing on immunocytochemistry (ICC), proteomic analysis, and AI-assisted diagnostics. ICC provides crucial information not only for subclassification but also for the assessment of relevant therapeutic biomarkers. Proteomic analysis enables comprehensive characterization of thousands of protein expression profiles simultaneously, using limited cytology materials. But its future use in routine diagnostics must be established and tested in clinical trials. Furthermore, digitalized cytological slides can be utilized for AI-based analyses, potentially reducing diagnostic workload and providing additional predictive information. Despite these advances, challenges remain regarding specimen preparation, standardization, and validation.

**Key Messages:**

The integration of morphology with appropriately validated molecular and computational methods has the potential to transform the framework of pulmonary cytology, extending its role from morphological diagnosis toward comprehensive and clinically significant characterization of pulmonary tumors.

## Introduction

The lung is one of the most frequent sites of primary and secondary malignant tumors, and pulmonary cytology is an integral part of daily cytological practice. Despite significant advances in diagnostics and treatment, lung cancer remains one of the major challenges of modern oncology. Worldwide, it still has the highest incidence of all types of cancer and represents the leading cause of cancer-related mortality [1]. Most patients are diagnosed with advanced-stage disease without surgical treatment options [2]. Notably, in a large proportion of patients, cytology specimens are the only source of tumor material [3]. In this era of precision medicine, comprehensive molecular testing by next-generation sequencing (NGS) for predictive gene alterations along with predictive immunohistochemistry has become a standard in patients with non-small cell lung cancer (NSCLC), and optimizing stewardship of all available tumor material is essential [4]. It is now undisputed that any cytological specimens, irrespective of fixation type, are principally equally suitable for biomarker analysis provided the assays have been adequately validated and a robust and rigorous quality assurance is in place [5]. Notably, liquid biopsy NGS from supernatants from cytological preparations and bronchioalveolar lavage effusions have shown high positive predictive values and high performance for mutation detection [6–8].

More than 70 years ago, the foundations of pulmonary cytology were laid by Papanicolaou and Cromwell, who pioneered the appreciation of cytology for the detection of lung cancer using sputum samples [9]. Over the past decades, (pulmonary) cytology’s applicability has broadened considerably. It is now used for diagnosis, classification, staging, and predictive marker analysis. For example, molecular testing by DNA- and RNA-based sequencing is now well established for cytology preparations. A guideline from the College of American Pathology, the International Association for the Study of Lung Cancer, and the Association for Molecular Pathology guideline stated that any type of cytology sample is suitable for molecular testing, as long as there is a high enough level of nuclear acid, tumor proportion, and appropriate validation of the methods [5].

The advantages of cytological samples are evident: (i) sampling can be performed minimally invasive, even from difficult-to-reach areas of the lung [10]; (ii) different preparation methods (smears, liquid preparations, and cell blocks) can be used; (iii) sample processing is quick; and (iv) DNA or RNA quality is excellent due to the lack of formalin fixation [11, 12].

These aspects have contributed to the rise of pulmonary cytology in recent years as a critical factor in clinical decision-making. The increasingly recognized importance of cytology is also reflected by the WHO Reporting System for Lung Cytopathology in 2022, which is one of the first organ-specific WHO cytology reporting systems (“purple books”) published so far [13].

This review gives a concise overview of the current role of pulmonary cytopathology, including the growing importance of immunocytochemistry (ICC), while also taking its limitations into account. Further, it highlights the opportunities and challenges of digital cytology entering the field as well as the potential application of proteomics and artificial intelligence (AI) that are likely to transform the broad field of cytology in the future.

## Growing Importance of Immunocytochemistry

The role of immunohistochemistry (IHC) has become indispensable not only for histological subclassification but also for therapeutic decision-making in pulmonary tumors. In patients with advanced-stage disease, however, the availability of tumor tissue is often limited. In addition, neoadjuvant chemoimmunotherapy for non-oncogene-driven disease and adjuvant EGFR and ALK targeted therapy, for the respective oncogene-addicted subgroups, have become standard treatment strategies for selected populations of patients with resectable early-stage lung cancer to locally advanced, necessitating upfront biomarker analysis on small specimens at the time of initial diagnosis. Given the relatively short turnaround time of ICC, its importance on conventional cytology specimens has been increasingly recognized, as cytology is the only available tumor material for diagnosis and predictive marker testing in a substantial proportion of lung cancer patients. While IHC markers using protocols established for histological samples are applicable to cell blocks, many markers are also applicable to ICC on Papanicolaou-stained cytology preparations fixed with alcohol-based methods. Due to preanalytical variability of cytology specimens with variable preparation types (including smears, cytospins, and liquid-based preparations), pre-fixatives, and staining protocols, ICC requires adaptation and separate validation of ICC protocols [14, 15] (Table 1).

**Table 1.** Examples of commonly used antibodies and corresponding clones used for immunocytochemistry in respiratory tract cytology [14, 16–24]

Purposes	Antigens	Clones
Cytological subtyping	TTF-1	8G7G3/1, SP141
​	p40	MR1244
​	Synaptophysin	SP11, SY38
​	Chromogranin A	LK2H10, MRQ-42
​	CD56	123C3.D5
​	INSM1	A-8
​	NUT	C52B1
​	SMARCA4	E8V5B, EPNCIR 111A
Identification of primary organs	Calretinin	SP65, Cal6
​	WT1	WT49L
​	Podoplanin	D2-40
​	Desmin	DE-R-11
​	EpCAM	Ber-EP4
​	Claudin4^a^	3E2C1
​	PAX8	EPR13510, MRQ-50
​	GATA3	L50-823
​	CDX2	DAK-CDX2, CDX-2-88
​	NKX3.1	EP356
Confirmation of neoplastic cell biomarkers	BAP1^a^	C-4
​	ALK	5A4, D5F3
​	ROS1	D4D6
​	NTRK	ERP17341
​	PD-L1	73-10, 22C3, SP263
​	MTAP^a^	2G4, MSVA-741R

^a^ These markers are not suitable for ethanol-fixed cytological preparations, but they are applicable to formalin-fixed cell blocks based on our own experience.

### Reliable Tumor Classification in Cytology Samples with ICC

Numerous lineage-specific markers used in the histological diagnosis of pulmonary tumors can also be applied to ICC. Immunostaining for TTF-1 and p40 plays a central role in subtyping non-small cell lung cancer, particularly when morphological features are ambiguous [13]. Neuroendocrine markers, such as synaptophysin, chromogranin A, and CD56, are helpful in confirming neuroendocrine differentiation. Mesothelial origin can be confirmed by application of calretinin, WT1, or podoplanin (D2-40). Together with MTAP, desmin and BAP1 deficiency, and lack of epithelial marker expression (e.g., BerEP4, Claudin4, or TTF1), may be used to distinguish metastatic carcinoma from mesothelioma in pleural fluid specimens. For excluding metastatic tumors to the lung, other specific lineage markers (e.g., CDX2, GATA3, PAX8 or NKX3.1) are also useful in ICC.

With advances in the molecular characterization of pulmonary tumors, some entities are defined by specific genomic alterations. One example is NUT carcinoma, characterized by *NUTM1* gene rearrangement resulting in NUT1 expression. Another is SMARCA4-deficient thoracic undifferentiated tumor, defined by loss of SMARCA4 expression. Immunostaining as surrogate markers for genomic alterations secures the diagnosis of these tumors by demonstrating marker positivity or loss, respectively [25–27].

### ICC as Driver of Therapeutic Decision Making and Continuous Expansion of the Predictive Repertoire

ICC also provides valuable information for guiding molecular testing and therapeutic decision-making. Rearrangements of genes such as *ALK, ROS1*, and *NTRK* represent druggable driver alterations. The respective protein expression is a surrogate for the molecular alteration and can be detected or screened for by ICC on cytologic preparations fixed with alcohol-based methods [14, 28–31] (shown in Fig. 1). A positive ICC result requires confirmation by fluorescence in situ hybridization (FISH) (shown in Fig. 1) or RNA-based NGS. This in contrast to ALK IHC, where a positive result is sufficient to trigger treatment, as laboratory developed ALK ICC assays are less standardized, and no commercial ALK ICC assays are available.

**Fig. 1.**
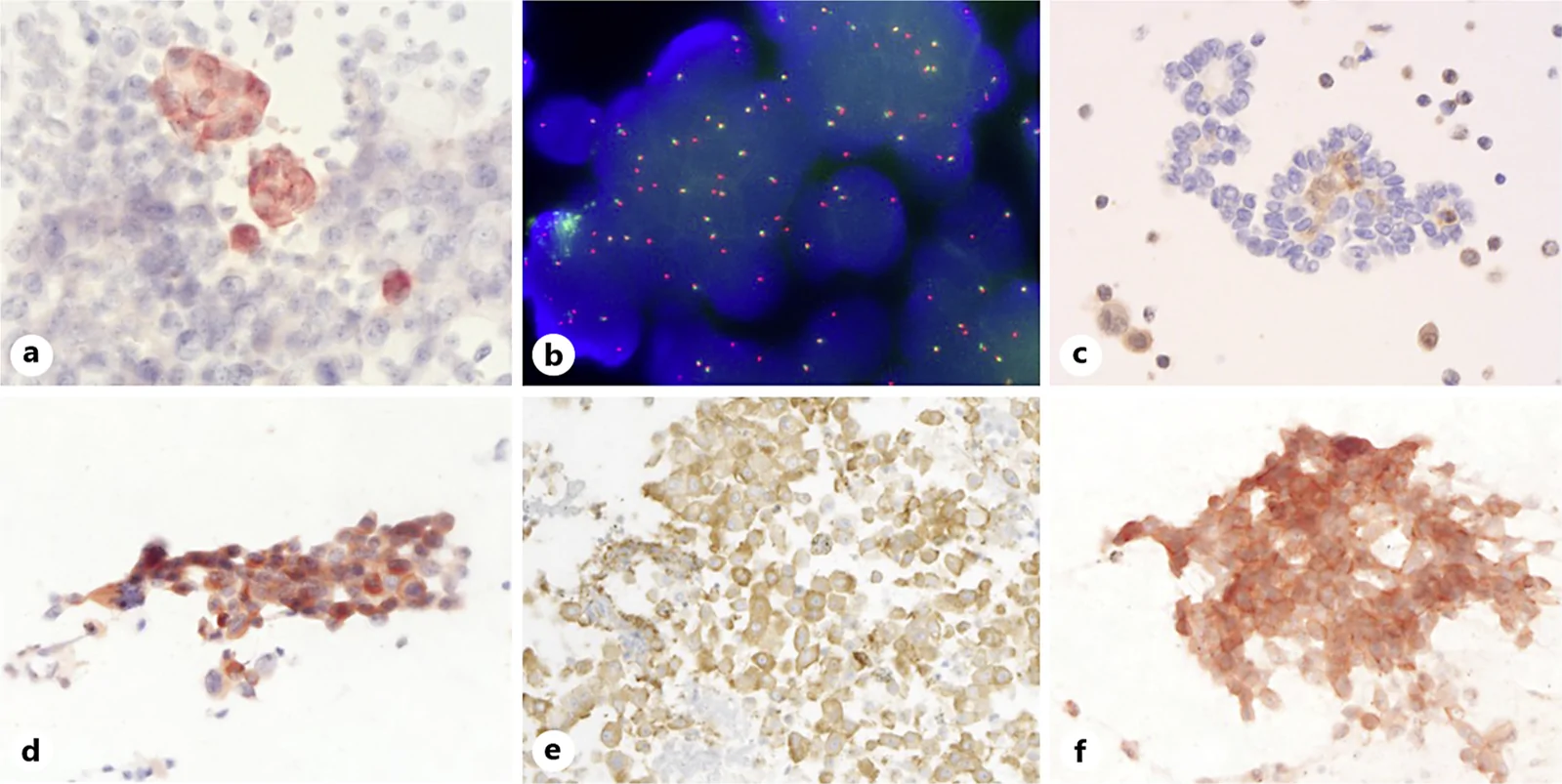
**a** Cytology smear of a pleural effusion showing an ALK positive adenocarcinoma by immunocytochemistry. **b** Corresponding ALK break-apart fluorescence in situ hybridization positive result with a single-red signal pattern. **c** Cell block of the same tumor showing MTAP deficiency by immunohistochemistry on cell block. **d** Cytology smear preparation of a bronchial secretion showing high PD-L1 expression by immunocytochemistry in another non-small cell carcinoma by tumor proportion score of 80%. **e** Cell block of a pleural effusion showing a non-small cell carcinoma with a pan-TRK positive immunohistochemistry in cell block. **f** Cytology smear from the same pleural effusion with corresponding pan-TRK positivity by immunocytochemistry. RNA-based next-generation sequencing confirmed a *TPM3::NTRK1* fusion.

PD-L1 expression can be evaluated on ethanol-fixed cytology preparations; however, careful interpretation is required when using the latter due to lack of standardization and the potential for membranous staining to appear cytoplasmic in cytological specimens, reflecting their three-dimensional structure and intact cell membranes [14, 15, 32] (shown in Fig. 1).

In addition, several emerging biomarkers may be applicable to ICC for patient stratification and screening for novel therapies in the era of precision medicine. MTAP, for example, has been reported not only as a diagnostic marker for mesothelioma but also as an emerging negative predictive biomarker for immune checkpoint inhibitors and as a potential therapeutic target of MTA-cooperative PRMT5 inhibitors [33, 34] in NSCLC. Because MTAP is consistently expressed in any benign cells, complete loss of expression (i.e., MTAP deficiency) is a significant finding. Based on our own experience, MTAP IHC is applicable to formalin-fixed cell blocks but not to ethanol fixed preparations (Fig. 1). Moreover, antibody-drug conjugates (ADCs), including an anti-HER2 reagent, trastuzumab deruxtecan, have become therapeutic options for advanced NSCLC [35]. Another ADC targeting c-MET, telisotuzumab vedotin, can also be used for treating advanced NSCLC with high c-MET expression [36]. The evaluation criteria for the expressional status of HER2 or c-MET have not been established in ICC yet. The promise and challenges of predictive IHC markers related to ADCs in cytology are addressed in a separate article by Md Ali et al. in this issue.

## Mass Spectrometry-Based Proteomics: Expanding Predictive Biomarker Analysis in Cytology

While ICC remains the cornerstone of protein-based predictive biomarker diagnostics in cytopathology, its analytical scope is inherently limited to a restricted number of predefined epitopes and available antibodies and limited samples. In contrast, mass spectrometry (MS)-based proteomics enables the simultaneous identification and quantification of thousands of proteins and signaling pathway components, providing substantially deeper insights into tumor biology, therapeutic response, and resistance mechanisms [37]. As proteins represent the functional endpoint of genomic and transcriptomic alterations and constitute the direct targets of most anticancer therapies, proteomic analyses provide biologically relevant information that complements genomic and transcriptomic profiling. Unlike antibody-based assays, whose analytical performance is affected by antibody specificity, accessibility of the epitope, or post-translational modifications that affect antibody binding, MS-based proteomics identifies and quantifies proteins through multiple independent peptide measurements. The resulting peptide coverage provides intrinsic quality control and orthogonal confirmation of protein identity, thereby increasing analytical robustness and reducing the likelihood of false-positive or false-negative results.

The expanding role of precision oncology has highlighted the need for multiplexed biomarker assessment beyond DNA and RNA analyses. Cytological specimens are particularly attractive for proteomic studies because they often contain highly enriched tumor cell fractions and frequently represent the only available diagnostic material in patients with advanced malignancies. In addition, cytological samples are commonly obtained from metastatic lesions or disseminated tumor deposits and may therefore reflect biologically selected tumor cell populations associated with invasion, metastasis, or treatment resistance. Nevertheless, the analysis of cytological specimens poses several challenges, including limited input material, fixation-related protein modifications, pre-analytical variability, and admixture with non-neoplastic cells. Notably, proteomic analyses can be successfully performed on samples prepared using fixation protocols implemented in routine cytopathology, including formalin-fixed cell blocks and cytologic preparations fixed with alcohol-based methods, without substantial loss of protein coverage [38].

For quantitative predictive biomarker analyses, accurate protein quantification generally requires highly enriched, ideally near-pure tumor cell populations, as admixed inflammatory or other non-neoplastic cells may mask or dilute tumor-specific proteomic signatures. Additional enrichment strategies may therefore be necessary, including laser capture microdissection of selected tumor cells followed by pooled proteomic analysis, or emerging single-cell proteomic approaches that enable characterization of individual tumor cells without interference from surrounding non-neoplastic cells [39].

Recent technological advances have substantially improved the feasibility of MS-based proteomics in clinical cytology. Innovations in sample preparation, nano-flow liquid chromatography, electrospray ionization tandem mass spectrometry (ESI LC-MS/MS), and high-sensitivity MS instrumentation now allow deep proteome coverage from minimal sample amounts [39, 40]. Notably, proteomic analyses can be performed from fewer than 100 tumor cells, and emerging workflows are increasingly approaching single-cell resolution. In particular, label-free data-independent acquisition strategies have improved reproducibility and quantitative completeness by increasing peptide coverage while reducing missing values compared with traditional data-dependent acquisition methods. In parallel, targeted proteomic assays enable highly sensitive and reproducible absolute quantification of clinically relevant proteins, offering a quantitative complement to conventional antibody-based approaches with relatively low per-sample measurement costs and short turn-around times.

Several studies have demonstrated the feasibility of proteomic profiling using fine-needle aspiration specimens, exfoliative cytology samples, liquid-based cytology preparations, and archived cell blocks (summarized in [41]). Importantly, residual material from routine diagnostic workflows can often be utilized without compromising morphological assessment.

Despite these advances, clinical implementation remains limited by the scarcity of large, clinically validated cohorts and the lack of prospective studies demonstrating clinical utility beyond established immunocytochemical, molecular, and cytogenetic assays. Future clinical studies are needed to directly compare MS-based proteomic biomarkers with current diagnostic standards, including antibody-based assays, next-generation sequencing, and FISH, to establish their added clinical value. In addition, analytical reproducibility and inter-laboratory robustness must be demonstrated across different diagnostic centers, sample preparation workflows, and mass spectrometry platforms to ensure reliable implementation in routine clinical practice. Nevertheless, continuing improvements in analytical sensitivity, robustness, and throughput suggest that proteomics may become an important component of future precision diagnostics, complementing conventional immunocytochemical and molecular approaches in cytopathology.

## Digitalization and Artificial Intelligence in Pulmonary Cytopathology

Digitalization and AI are beginning to reshape cytopathology with a rapid transition from conceptual innovation and research to an increasing part of routine cytopathology workflows.

Pulmonary cytology is a still relatively underdeveloped application area compared with lung histopathology and gynecological cytology. Fine needle aspiration, bronchial cytology, and pleural fluid analysis are central to the diagnosis and staging of lung cancer, yet digital and AI tools for these specimen types have only recently begun to mature.

### Digital Cytopathology: General Developments and Relevance to Pulmonary Cytology

Digital cytology is built on whole slide imaging (WSI) of cytology preparations, coupled with digital workflows, telecytology, and computational image analysis. Cytological material poses greater technical challenges than histology by showing 3D and overlapping clusters, variable thickness of the material to analyze, and the need for continuous refocusing to appreciate nuclear detail and background. These factors have contributed to slower adoption in cytology than in surgical pathology [42, 43].

There are some key advantages of digital cytology, like remote consultation and telecytology; the creation of large digital archives for education, quality assurance, research, and foundational infrastructure for AI model development [43–45]. At the same time, there are still unresolved issues like scanning speed and efficiency for multi-z imaging, data volume and storage, interoperability with laboratory information systems, and limited routine diagnosis data.

While digitalization of gynecological cytology (Pap-tests) started decades ago and today is used in routine diagnostics in some institutions (screening of cervical cancer/Pap-test), digitalization of non-gyn cytology is in a much earlier stage of development. A global survey shows increasing but heterogeneous implementation, with many laboratories in exploratory or pilot phases [46]. A 5-year experience from Bychkov et al. demonstrates that full integration of cytology into a routine digital pathology workflow, including non-gynecologic material, is feasible with stepwise implementation and robust validation [47].

Pulmonary cytology particularly depends on high-quality z-stack imaging or multiple focus points to reproduce real-time focusing. Multi-plane scanning and extended focus algorithms, as well as proper stack-compression technologies, aim to provide optimal focus at manageable data volumes. These are crucial enablers for large-scale digitization of respiratory cytology and, by extension, AI training and deployment.

### AI in Pulmonary and Respiratory Cytology

Emery et al. provide a comprehensive review of AI in cytopathology, documenting a rapid expansion from cervical screening into non-gynecologic areas including urine, thyroid, effusions, breast, and lung [42]. Early cytology AI largely addressed binary questions (benign vs. malignant). More recent studies extend to tumor subclassification, and – more experimentally – morphology-based molecular prediction.

An umbrella review [43] and other AI-focused series in Acta Cytologica [48–54] emphasize common barriers: lack of large, open, well-annotated datasets; heterogeneous preparations and staining; less matured or standardized reporting systems and scoring frameworks for many non-gynecologic cytology; and the intrinsic difficulty of modeling multilayered, 3D cytology clusters with predominantly 2D algorithms.

Nonetheless, multiple studies across specimen types now show that deep learning can match or exceed expert performance and that AI assistance can improve accuracy and reduce inter-observer variability [42, 43, 55]. A pilot work applying AI within existing classification systems (e.g., The Paris System) suggests that computational tools can be integrated into established diagnostic frameworks [56].

Within this broader field, pulmonary cytology remains relatively under-represented despite its clinical centrality. AI has been examined extensively in lung histopathology but relatively few studies in lung cytology [42]. Contributing factors include lack of public lung cytology datasets, dependence on architectural clues for certain diagnoses, and the morphologic heterogeneity of respiratory specimens. Despite these challenges, an emerging set of studies demonstrates that AI can be successfully applied for various purposes.

Although focused on metastatic breast carcinoma, one work on malignant pleural effusion cytology is methodologically informative for thoracic practice [57]. This underscores a recurrent pattern: stand-alone AI can be strong, but its most compelling value lies in interactive human–AI workflows. Even a multicenter study presents AI in respiratory cytology as a decision-support tool that enhances, rather than replaces, expert human interpretation [58].

Another study presented convoluted neural networks (CNNs) to distinguish small cell lung carcinoma (SCLC) from large cell neuroendocrine carcinoma (LCNEC) in cytology and associated cell block sections [59]. This proof-of-concept report indicates that AI can capture subtle chromatin and cytologic differences in high-grade neuroendocrine neoplasms.

Morphology-based molecular prediction is perhaps the most forward-looking application. CNNs trained in lung cytology could predict KRAS/EGFR status and ALK rearrangements with high accuracy at the cell level [42, 60]. This parallels histology-based work in morphology-to-genotype prediction and suggests that AI-enhanced cytology may eventually provide preliminary molecular inference or triage when tissue is limited.

AI was also applied to segment malignant clusters in respiratory smears for use in ROSE [42]. These systems aim to support adequacy assessment and rapid triage during bronchoscopic and transthoracic FNA procedures. By automatically highlighting malignant clusters and displaying probability maps, they may shorten procedures, improve sampling adequacy, and structure interactions between cytopathologists and interventional pulmonologists.

### Validation, Implementation, and Remaining Challenges

To date, there are no widely available, regulatory-cleared AI systems specifically targeting lung cytology. Commercial efforts remain focused on cervical cytology and on generic platform technologies that could later host organ-specific models.

Digital cytology validation studies in urine, effusions, and FNA specimens show that WSI can be as good as analog microscopy for primary diagnosis when cytology-specific issues are addressed [61, 62]. The same principles are valid for safe AI implementation. Guidance from the American Society of Cytopathology (ASC) Task Force and European Society of Digital and Integrative Pathology (ESDIP) on digital pathology validation and workflow design is directly relevant to pulmonary cytology [44, 45, 63].

Most pulmonary cytology AI studies are single-country or single-institution with limited external validation. Public, well-annotated respiratory cytology datasets are needed [64]. Future systems will need to integrate cytomorphology with immunocytochemistry and molecular testing, building comprehensive AI models [53, 65]. Some authors even speculated that AI might represent a “third wave” of revolutionizing cytopathology after immunocytochemistry and molecular diagnostics that aid personalized medicine [48].

Ensuring transparency, robust validation, and a clearly defined augmentative role for AI is essential. Even though major challenges like proper validation on large datasets, integration with precision oncology, and regulatory and ethical issues remain, today we know that AI contributes to better and faster diagnosis and aids cytopathologists in everyday work instead of replacing our profession.

### Future Perspectives of Digitalization and Artificial Intelligence in Cytology

Digitalization and AI are beginning to change pulmonary cytopathology, moving from proof-of-concept studies toward clinically relevant tools. As a large, multicenter work in respiratory cytology and pleural effusions demonstrates, AI/deep learning can reach or exceed expert performance and, importantly, meaningfully enhance human diagnostic accuracy and interobserver agreement [57, 58]. Subspecialized models for molecular prediction point toward an AI-enabled, minimally invasive, precision medicine paradigm in thoracic oncology.

To realize this potential, the field will require robust digital infrastructure, multicenter datasets, carefully designed validation, and sustained collaboration among cytopathologists, computer scientists, industries, and regulators. Properly developed and implemented, digital cytopathology and AI will not replace the pulmonary cytopathologist but instead extend diagnostic capabilities, standardize practice, and improve outcomes for patients with lung cancer and other thoracic diseases.

## Challenges in Implementing Novel Technologies to Clinics

Although recent advances in pulmonary cytology have introduced a variety of innovative technologies, several challenges remain before these approaches can be fully integrated into routine clinical practice.

Unlike histological specimens, from which multiple similar sections can be prepared by additional cutting, cytological slides are often unique and non-reproducible in nature. Therefore, when morphological evaluation must be combined with sample-consuming analyses, appropriate archiving strategies, such as scanning slides as virtual images, may be required.

Another challenge inherent to cytological specimens is the wide variety of fixation and staining methods used in routine practice, even among samples derived from the same organ. The optimal sample quality, quantity, and fixation method differ depending on the analytical modality. For example, modification of the IHC protocol established for formalin-fixed histology samples is required for ICC on ethanol-fixed cytological specimens. Since protein structure is differently affected by fixation methods, the way of antigen retrieval and/or selection of antibody clones should be reconsidered and validated [66]. On the other hand, since formalin fixation impairs nucleic acid quality, ethanol-fixed cytology preparations are superior for DNA- and RNA-based sequencing, and less tumor cells and/or a lower tumor cell proportion might be acceptable [67, 68]. Optimization of sample preparation procedures and standardization of staining or analytical protocols are desirable. Furthermore, the development of AI-assisted diagnostic systems capable of handling diverse cytological conditions may require training using large numbers of samples prepared under various conditions.

There also remains a gap between technological innovation and practical clinical implementation. Although genetic and proteomic testing are applicable to cytological samples, these technologies often require expensive equipment and highly specialized expertise, limiting their availability to selected institutions. In some situations, centralized testing strategies may therefore be unavoidable.

In addition, caution is warranted when incorporating novel technologies, such as comprehensive proteomic analysis and AI, into routine diagnostic practice, especially when their clinical reliability has not yet been fully established. For example, some AI-assisted quantification algorithms for PD-L1 tumor proportion score in histological NSCLC slides have already been Conformité Européenne-in vitro diagnostic marked and commercially available [69, 70]; however, application to cytological slides requires further validation.

## Conclusion

As the technologies introduced in this review continue to evolve and become applicable to clinical practice, the role of cytology in respiratory medicine is expected to expand in various directions (Fig. 2). There is a common understanding that cytological material is well suited for broad biomarker testing to direct lung cancer management, and its importance will likely expand.

**Fig. 2.**
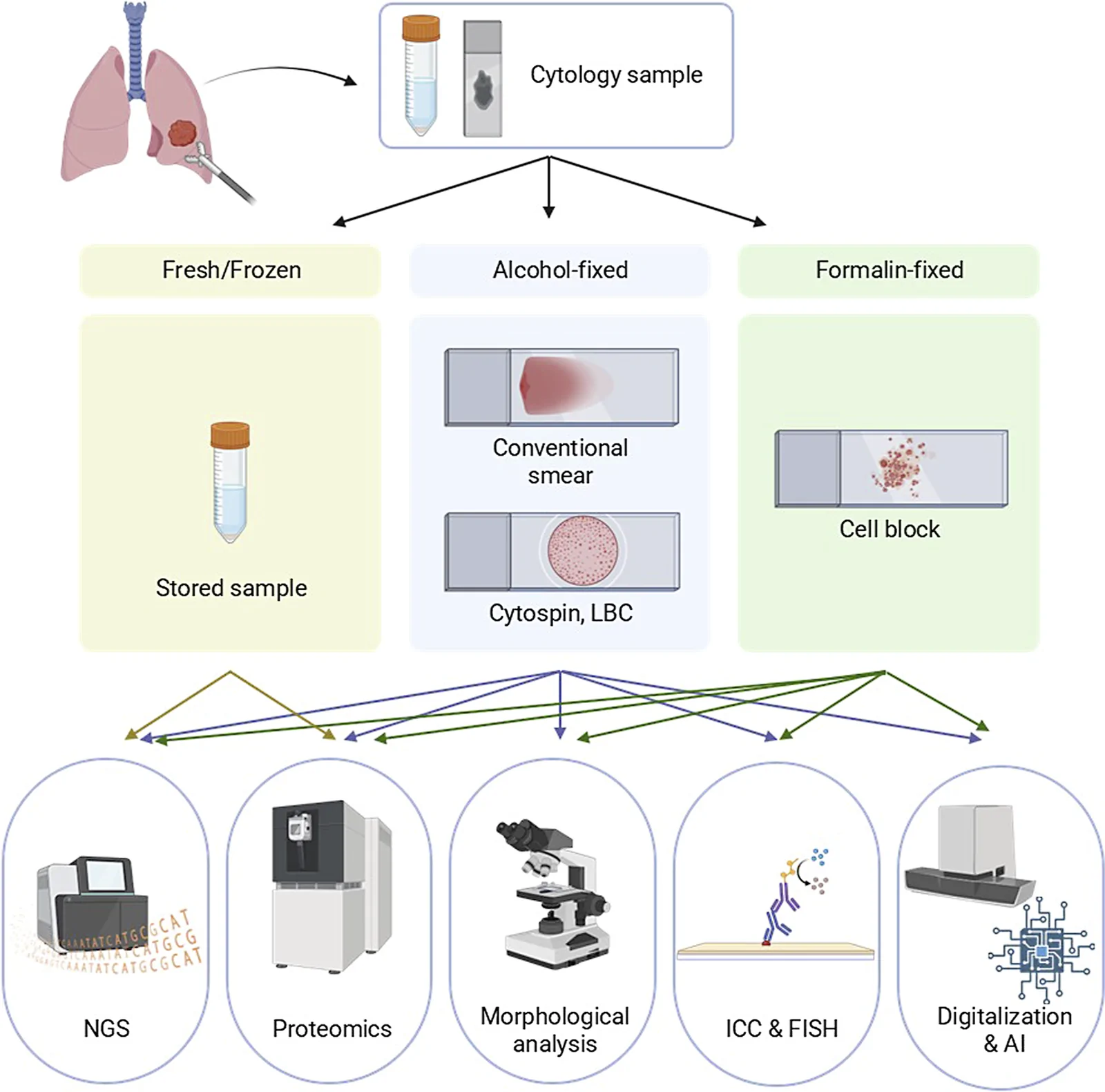
Emerging diagnostic and predictive methods in cytology. Created in BioRender [71]. AI, artificial intelligence; FISH, fluorescence in situ hybridization; ICC, immunocytochemistry; LBC, liquid-based cytology, NGS, next generation sequencing.

Future cytopathological practice may therefore require not only expertise in morphology but also a comprehensive understanding of the characteristics and limitations of these ancillary diagnostic techniques. Emerging methodologies, including proteomics and AI, will require adaptation of the workflow in the cytology laboratory and may improve diagnostic accuracy and transform cytology practice.

## Acknowledgments

For correction of grammatical errors and enhancements of readability of the draft, generative AI (ChatGPT, https://chat.openai.com/chat) was used.

## Conflict of Interest Statement

L.B. reports receiving grants from Roche, Novartis, Boehringer Ingelheim, Thermo Fisher, and AstraZeneca; consulting fees from Bristol-Myers Squibb, Eli Lilly, Bayer, Amgen, Takeda, Merck Sharp and Dohme, Janssen, and Systems Oncology; and honoraria from Thermo Fisher, Takeda, Janssen, and AstraZeneca. S.S.P. reports receiving grants from consulting fees from Merck Sharp and Dohme and AstraZeneca; honoraria from Abbvie AG. L.B. and S.S.P. were members of the journal’s Editorial Board at the time of submission. The other authors have no conflicts of interest to declare.

## Funding Sources

This study was not supported by any sponsor or funder.

## Author Contributions

J.K., M.M.B., and L.B.: writing – original draft and editing the manuscript. N.D. and E.D.-R.: writing – original draft. S.S.P. and O.C.: writing – review and editing. All authors have reviewed the manuscript and agreed to the submitted version of the manuscript.
